# Redirecting barley breeding for grass production through genome editing of *Photoperiod-H1*

**DOI:** 10.1093/plphys/kiae075

**Published:** 2024-02-16

**Authors:** Daisuke Tezuka, Huikyong Cho, Hitomi Onodera, Qianyan Linghu, Takeshi Chijimatsu, Masahiro Hata, Ryozo Imai

**Affiliations:** Institute of Agrobiological Sciences, National Agriculture and Food Research Organization (NARO), Tsukuba 305-8604, Japan; Institute of Agrobiological Sciences, National Agriculture and Food Research Organization (NARO), Tsukuba 305-8604, Japan; Institute of Agrobiological Sciences, National Agriculture and Food Research Organization (NARO), Tsukuba 305-8604, Japan; Institute of Agrobiological Sciences, National Agriculture and Food Research Organization (NARO), Tsukuba 305-8604, Japan; SASAKI FOOD Co., Ltd, Bungotakada 879-0615, Japan; SASAKI FOOD Co., Ltd, Bungotakada 879-0615, Japan; Institute of Agrobiological Sciences, National Agriculture and Food Research Organization (NARO), Tsukuba 305-8604, Japan; Faculty of Life and Environmental Sciences, University of Tsukuba, Tsukuba 305-8572, Japan

## Abstract

Genome editing enables precise modification to harness an elite grain-producing barley (*Hordeum vulgare* L.) cultivar for grass production.

Dear Editor,

Crop genome editing provides a substantial advantage over conventional breeding by enabling precise improvements in elite varieties. Among cereals, barley (*Hordeum vulgare* L.) holds the fourth position in global importance and has wide applications in malting and brewing. In regions like East Asia, barley grains have traditional culinary uses, being directly cooked as steamed barley, roasted for tea, or fermented for products like miso and soy sauce. Notably, the recent health trends have amplified interest in young barley grass as a functional health food. Owing to its rich content of vitamins, fibers, and flavonoids, barley grass is processed into green juice powder (Havlíková et al. 2014). This green powder has demonstrated effectiveness in antiulcer, hypolipidemic, and antidiabetic activities (Yu et al. 2003; Yoshizawa et al. 2004; Takano et al. 2013). In Japan, the rainy season frequently precedes the harvest season, making preharvest sprouting a challenge for grain yield. To address this, the elite varieties cultivated exhibit early heading traits. However, these traits negatively impact young barley grass yield. Specifically, the emergence of young spikes diminishes the commercial value of the grass. Current climatic shifts, attributed to global warming, have accelerated and destabilized spike development, decreasing grass yields. A shift in breeding approaches, focusing on late heading traits in contemporary varieties, is crucial to maintain consistent grass yields. Our previous work introduced a transgene-free genome editing technique, in planta particle bombardment-ribonucleoprotein (iPB-RNP), in wheat (*Triticum aestivum* L.) (Kumagai et al. 2022). This technique employs CRISPR/Cas9 ribonucleoproteins introduced directly into shoot apical meristems (SAMs), specifically targeting subepidermal cells that are potential germ-line cells (Goldberg et al. 1993; Reiser and Fischer 1993). The beauty of the iPB-RNP method is its genotype independence, allowing its application across a spectrum of elite wheat varieties (Liu et al. 2021; Kumagai et al. 2022). Its extension to crops other than wheat has been uncharted until now. In this study, we adapted the iPB-RNP method for barley and showcased its potential in addressing climatic challenges via genome editing in a select barley variety.

Our editing targeted the *Photoperiod-H1* (*Ppd-H1*) gene, encoding a pseudo-response regulator (*PRR*) governing photoperiod sensitivity. Notably, natural variations in *Ppd-H1*, resulting in amino acid substitutions in the CCT domain, lead to diminished photoperiod sensitivity and subsequent late flowering under long-day (LD) conditions (Turner et al. 2005). Since such mutant alleles are recessive and the homoeologous mutants in wheat also demonstrate late flowering (Shaw et al. 2013), disrupting the *Ppd-H1* gene was anticipated to induce late flowering. Using 2 guide RNAs targeting the third exon of *Ppd-H1* (Fig. 1A), and preassembled with recombinant SpCas9 protein, the RNP complex was introduced into the SAMs of a Japanese elite cultivar ‘Nishinohoshi’ via particle bombardment (Supplementary Materials and Methods). Evaluating around 600 E_0_ plants grown from these SAMs using cleaved amplified polymorphic sequences (CAPS) assays, we identified mutations at each target site. Notably, almost all CAPS-positive plants presented with a 1 base pair insertion or deletion, indicating potential loss of Ppd-H1 function due to a frameshift (Fig. 1B, Supplementary Figs. S1 and S2 and Table S1). Given that E_0_ mutants produced using the iPB technique are chimeric (Kumagai et al. 2022), the ultimate plant selection was conducted with the E_1_ generation. We successfully isolated 2 genome-edited homozygous lines, NH7-6 and NH3-2, from 16 E_0_ plants (Fig. 1, C and D, Supplementary Fig. S3 and Table S1). The summary of the *ppd-H1* mutant screening is depicted in Fig. 1E. The mutation occurrence rates in these plants were consistent with those observed in wheat (Kumagai et al. 2022).

**Figure 1.**
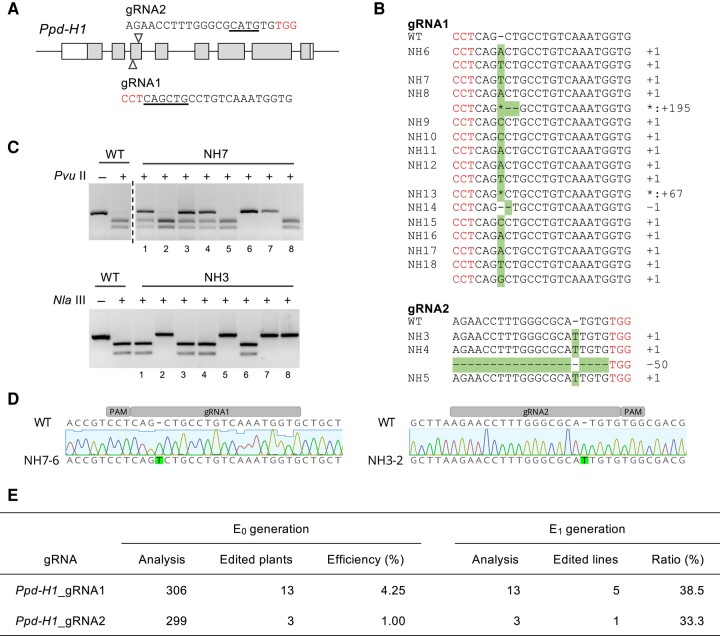
Creation of the *ppd-H1* mutants by *in planta* particle bombardment. **A)** Schematic representation of the *Ppd-H1* gene with gRNA design. The white and gray boxes signify untranslated regions and exons, respectively. Red characters within the gRNA sequences denote the protospacer adjacent motif (PAM), while underlines highlight the restriction enzyme sites intended for CAPS assays. **B)** Noted genome-edited mutations in each CAPS-positive E_0_ plant. Mutations introduced by genome editing are highlighted. **C)** Genotypic analysis of E_1_ offspring derived from E_0_ NH7 and E_0_ NH3 plants. “−” and “+” denote digestion with and without restriction enzymes, respectively. **D)** Detailed sequence evaluation of genome-edited E_1_ homozygous plants, specifically NH7-6 and NH3-2. **E)** Overview of the genome editing experiments.

To discern the flowering phenotype of the *ppd-H1* mutants, lines NH7-6 and NH3-2 were cultivated in controlled growth chambers with LD conditions (16 h light/8 h dark). As anticipated, the heading date for the *ppd-H1* mutants was delayed by ∼40 d compared with the wild type (Fig. 2, A and B). Notably, green grass production just before spike development was roughly 17 times higher than that of the wild type (Fig. 2C). This implies that the *ppd-H1* mutant lines can potentially circumvent premature heading, extending the grass harvest window and promoting stable production. Changes in gene expression were analyzed with wild-type and mutant lines. Within a loop of morning active genes, a homolog of Ppd-H1, AtPRR7, has been shown to downregulate *CIRCADIAN CLOCK ASSOCIATED 1* (*AtCCA1*) which in turn positively regulates *AtPPR7* (Farré et al. 2005; Nakamichi et al. 2010). Our observations in the *ppd-H1* mutants indicate that mRNA of the barley *CCA1* (*HvCCA1*) gene tends to accumulate at higher levels during 0 to 8 h in the *ppd-H1* mutants (Fig. 2D, Supplementary Tables S1 and S2), whereas *Ppd-H1* mRNA tends to accumulate at higher levels in the *ppd-H1* mutants between 4 and 8 h (Fig. 2D, Supplementary Tables S1 and S2). These data support a functional similarity between Ppd-H1 and AtPRR7 (Kusakina et al. 2015). In addition, we also noted a similar level of expression for barley *TIMING OF CAB EXPRESSION 1* (*HvTOC1*) between wild type and the mutants. Expression analyses of flowering genes revealed diurnal expression patterns of barley GIGANTEA (*HvGI*) and *CONSTANS* (*HvCO1* and *HvCO2*) similar across wild-type and mutant plants. The barley *FLOWERING LOCUS T* (*HvFT1*) expression in the *ppd-H1* mutants remained significantly reduced, ranging from 0.05% to 0.67% of the wild type, yet exhibited circadian rhythms akin to the wild type (Fig. 2D, Supplementary Fig. S4 and Tables S1 and S2). This suppressed *HvFT1* expression in *ppd-H1* mutants persisted throughout the vegetative growth period (Fig. 2E, Supplementary Table S1). Such findings align with the late flowering phenotype of the *Ppd-H1* natural variants (Turner et al. 2005), suggesting that these variants may indeed be functionally suppressed. Apart from grass yield benefits, grain yield metrics, encompassing spike count, seed quantity, and aggregate seed weight, were elevated compared with the wild type, possibly stemming from the extended cultivation window (Supplementary Fig. S5).

**Figure 2.**
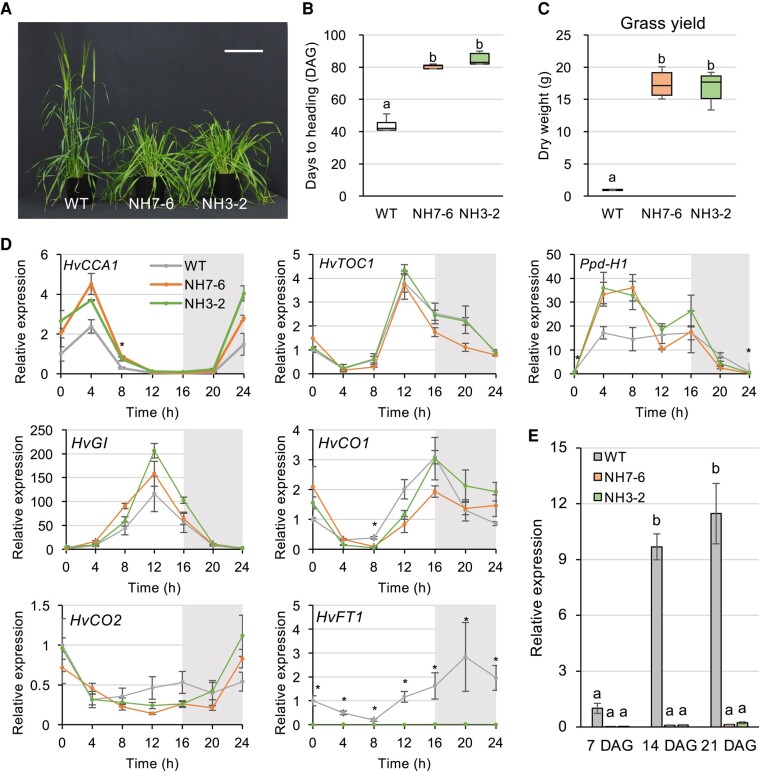
Phenotype of the *ppd-H1* mutants and expression analyses of flowering genes. **A** and **B)** Examination of the heading date in wild-type plants vis-à-vis the *ppd-H1* mutants. Both wild-type and *ppd-H1* mutant plants were cultivated under LD conditions (16 h of light followed by 8 h of darkness). Center lines in boxplots show the medians; box limits indicate the 25th and 75th percentiles; whiskers are the minimum and maximum of all data (*n* = 6). Distinct letters indicate significant differences as determined by Tukey's test (*P* < 0.01). The scale bar represents 20 cm. DAG, days after germination. **C)** Grass production assessment of *ppd-H1* mutants. The aboveground segments of plants, taken just prior to inflorescence evolution, underwent drying at 85 °C over a 7-d period before weighing. Center lines in boxplots show the medians; box limits indicate the 25th and 75th percentiles; whiskers are the minimum and maximum of all data (*n* = 6). Distinct letters indicate significant differences as determined by Tukey's test (*P* < 0.01). **D)** Circadian patterns of flowering gene expressions in *ppd-H1* mutants. Total RNA was procured from mature leaves of 14-d-old plants grown in LD conditions (16 h light/8 h dark intervals). The graph’s shaded area marks the dark phase. The provided data are averaged with the Se based on 4 replications (*n* = 4). An asterisk indicates a time point at which both *ppd-H1* mutants demonstrated significant differences compared with the wild type, as determined by Tukey's test (*P* < 0.05). A detailed summary of the statistical analysis is presented in Supplementary Table S2. Specific primers employed for the RT-qPCR can be found in Supplementary Table S1. **E)***HvFT1* gene expression observed during the vegetative growth phase. The total RNA was isolated from mature leaves at sequential time points: 7, 14, and 21 DAG. Data delineates the mean ± Se with 4 replicates (*n* = 4). Distinct letters signify remarkable differences based on Tukey's test (*P* < 0.01).

Conclusively, while barley breeding in Japan has historically gravitated toward early heading traits to mitigate grain yield losses from preharvest sprouting due to regional climatic nuances, this very trait hinders young barley grass production. Our research marks a successful demonstration of a genotype-independent, nonculture genome editing technique tailored for barley, effectuating precise modifications to heading characteristics in an elite variety. This case exemplifies the transformative power of genome editing in repurposing an elite grain harvesting variety for grass harvesting.

## Supplementary Material

kiae075_Supplementary_Data

## Data Availability

All data that support the findings of this study are available in the main paper and Supplemental data.

## Supplementary data

The following materials are available in the online version of this article.

**Supplementary Figure S1**. E_0_ plant screening.

**Supplementary Figure S2**. Examination of the *Ppd-H1* gene in E_0_ mutants.

**Supplementary Figure S3**. E_1_ plant genotyping.

**Supplementary Figure S4**. Diurnal expressions of *HvFT1* in the *ppd-H1* mutants.

**Supplementary Figure S5**. Assessment of grain yield of the *ppd-H1* mutants.

**Supplementary Table S1**. Primers used in this study.

**Supplementary Table S2**. Summary of statistical analysis on diurnal expression.
