# Supplementary material for: Redirecting barley breeding for grass production through genome editing of Photoperiod-H1
Source: Plant Physiol. 2024 Feb 16;195(1):287–90. doi: 10.1093/plphys/kiae075 (PMC11060677; doi:10.1093/plphys/kiae075)
Supplement: kiae075_Supplementary_Data [file kiae075_supplementary_data.pdf]

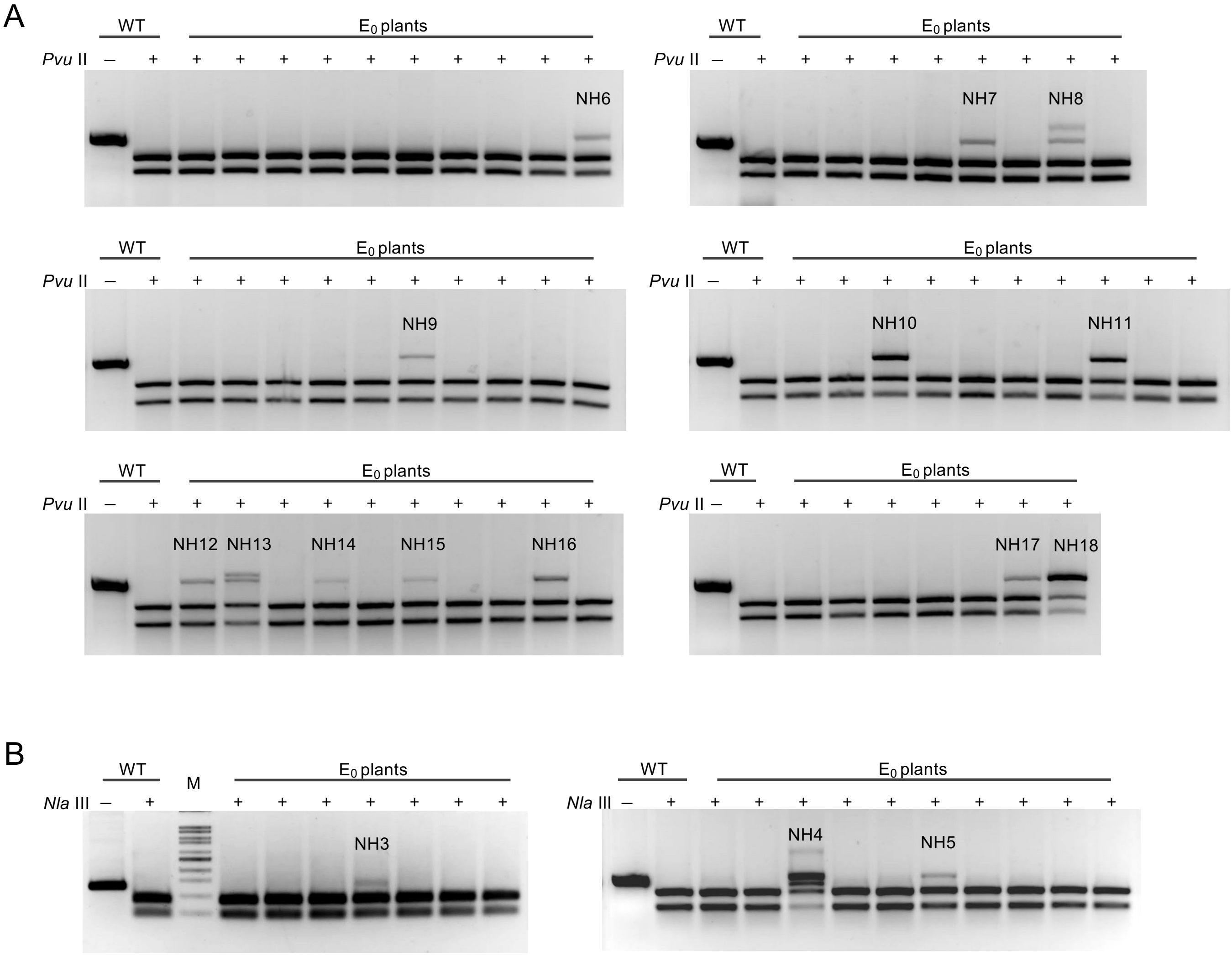

Supplemental Figure S1. E_0 plant screening

The fifth leaf of each E_0 plant was isolated for genotyping via CAPS. *Ppd-H1* fragments containing either gRNA1 or gRNA2 sequences were amplified with gene-specific primers detailed in Supplemental Table S1. Subsequently, these fragments underwent digestion using *Pvu* II or *Nla* III enzymes to detect mutations in the gRNA1 (A) or gRNA2 (B) target sequences. Plants displaying undigested fragments on an agarose gel were selected. "–" and "+" denote digestion with and without restriction enzymes, respectively.

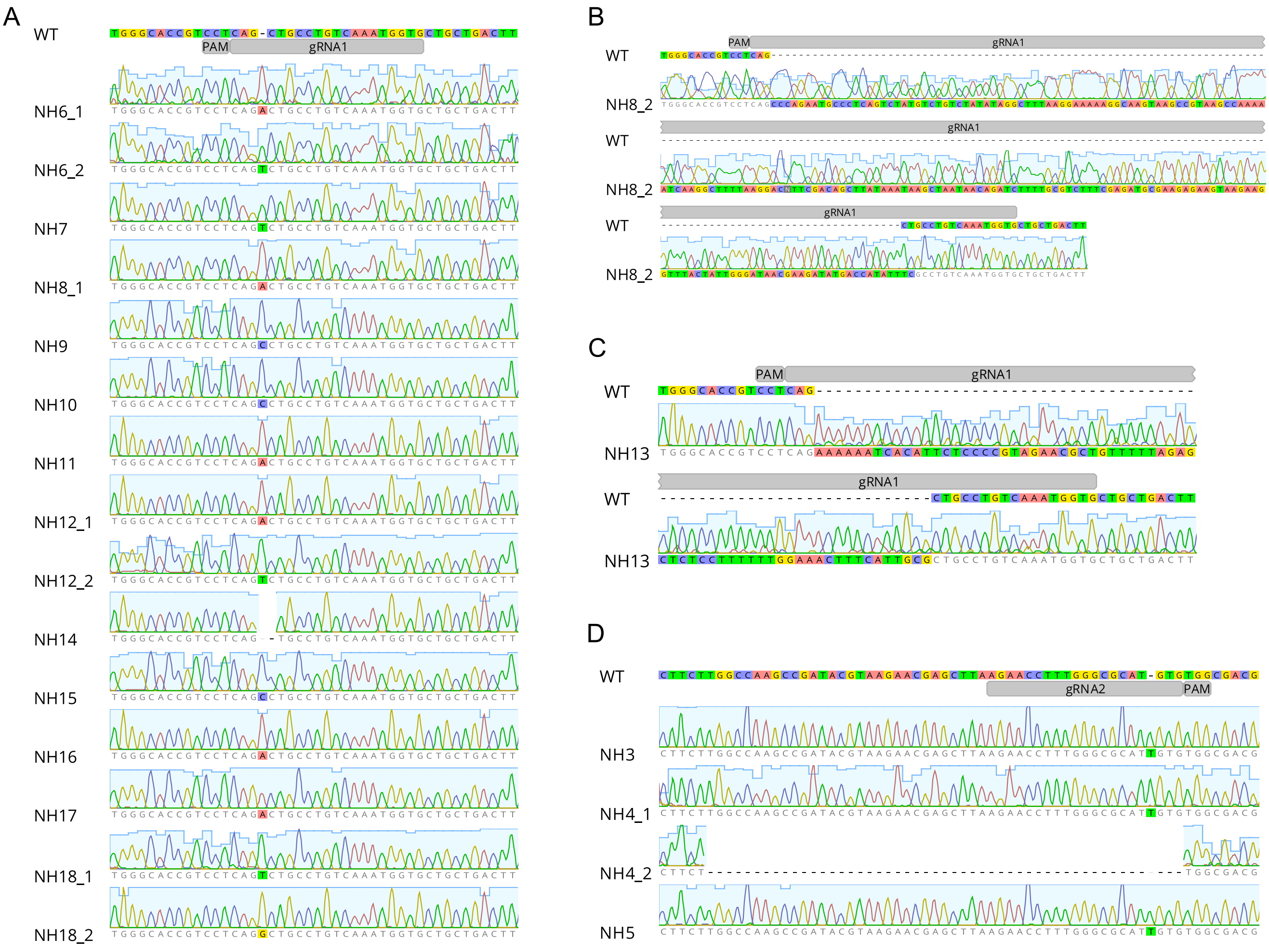

Supplemental Figure S2. Examination of the *Ppd-H1* gene in E_0 mutants

The undigested fragments derived from the CAPS analysis (as presented in Supplemental Figure S1) were cloned. Sanger sequencing was employed to discern the *Ppd-H1* partial sequences from each E_0 mutant. (A) One base-pair insertion or deletion detected at the gRNA1 target sequence. (B, C) Long insertion detected at the gRNA1 target sequence. Note that insertion sequences are barley mitochondrial DNA. (D) Mutations detected at the gRNA2 target sequence.

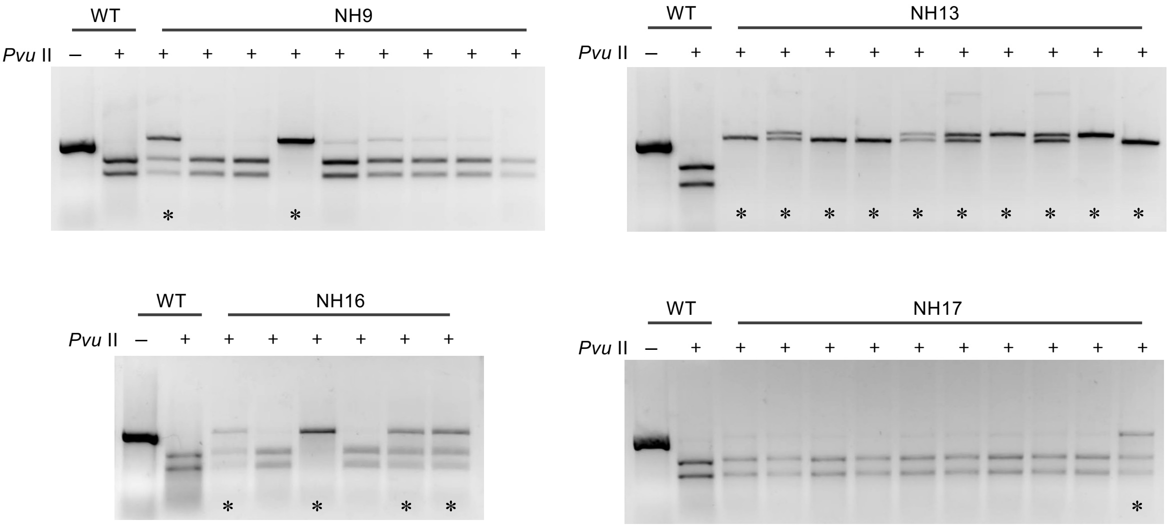

Supplemental Figure S3. E_1 plant genotyping

DNA from E_1 seedlings underwent CAPS analysis. E_1 mutants originated from NH9, 13, 16, and 17, in addition to NH3 and NH7, as illustrated in Figure 2C. "–" and "+" denote digestion with and without restriction enzymes, respectively. Asterisks indicate E_1 genome-edited mutants based on restriction digestion.

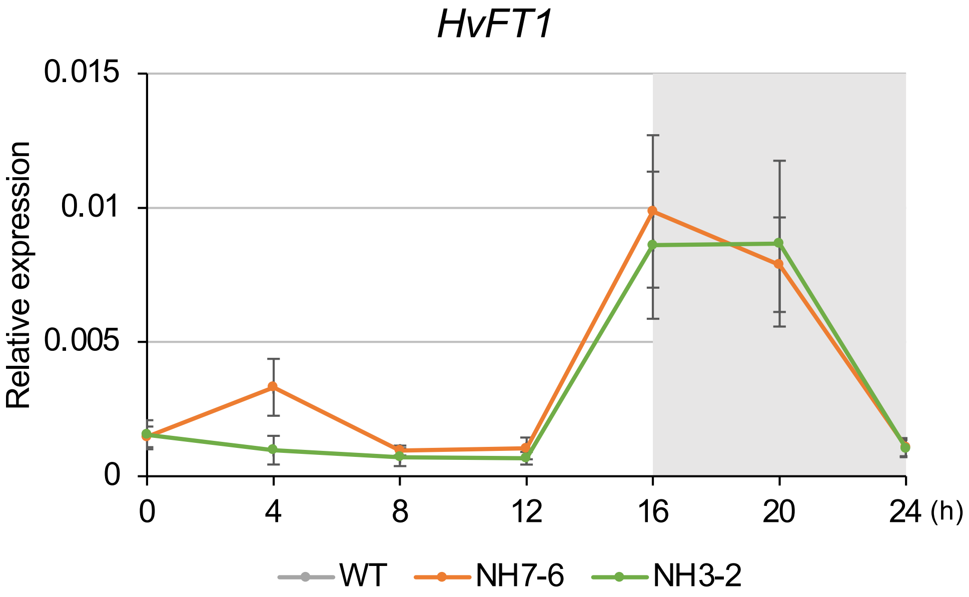

Supplemental Figure S4. Diurnal expressions of *HvFT1* in the *ppd-H1* mutants
Enlargement of Figure 2D. Gray background in graph indicates dark condition. The data represent the mean ± SE ($n = 4$).

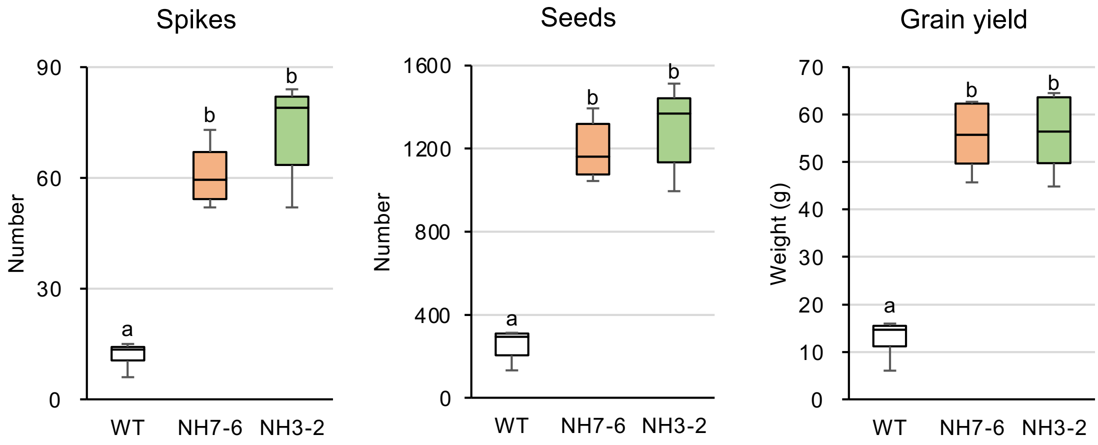

Supplemental Figure S5. Assessment of grain yield of the *ppd-H1* mutants
The *ppd-H1* mutants were cultivated under long-day (LD) conditions (16h light/8h dark intervals). Parameters such as spike count, seed count, and per-plant grain weight were recorded. Center lines in boxplots show the medians; box limits indicate the 25th and 75th percentiles; whiskers are the minimum and maximum of all data (n = 6). Distinct letters signify remarkable differences based on Tukey's test ($p < 0.01$).

Supplemental Table S1. Primers used in this study

Name	Fw sequence (5' -> 3')	Rv sequence (5' -> 3')
gRNA1 synthesis	TAATACGACTCACTATAGCACCATTTGACAGGCA	TTCTAGCTCTAAAACCAGCTGCCTGTCAAATGGT
gRNA2 synthesis	TAATACGACTCACTATAGAGAACCTTTGGGCGCAT	TTCTAGCTCTAAAACCACATGCGCCCAAAGGTTCT
gRNA1 screening	CGACCTTGTTCTCACAGAGG	TGGCACTTCCACTACCACTG
gRNA2 screening	ATGGGCACTGTCCTCAGC	TTGACCGAGTAAAAGATGCAC
HvGI RT-qPCR	TCAGTTAGAGCTCCTGGAAGT	GGTAGTTTGGGCTTTGGATG
HvCO1 RT-qPCR	GGGGCAGAGCAGGCTGCCTC	TGGCTTCTCTCTCCTTGGAGC
HvCO2 RT-qPCR	CATCACTTGTGACCCAAGACC	CTATAGTTCCATAATTGCTCC
HvFT1 RT-qPCR	CCAACCTTAGAGAGTATCTCCACT	CCCTGGTGTTGAAGTTCTGG
Ppd-H1 RT-qPCR	GATGGATTCAAAGGCAAGGA	GAACAATTGGCTCCTCCAAA
HvCCA1 RT-qPCR	CCTGGAATTGGAGATGGAGA	TGAGCATGGCTTCTGATTTG
HvTOC1 RT-qPCR	GGTCAGTTTGTTAGGCAGGCAAG	GGTGAAGAAACCAACTCAACGTCC
HvActin RT-qPCR	CGTGTTGGATTCTGGTGATG	AGCCACATATGCGAGCTTCT

Supplemental Table S2. Summary of statistical analysis on diurnal expression

Gene	Time point (h)	Tukey's test ($P < 0.05$)			Tukey's test ($P < 0.01$)		
		WT	NH7-6	NH3-2	WT	NH7-6	NH3-2
HvGI	0	–	–	–	–	–	–
	4	a	b	ab	–	–	–
	8	a	b	ab	–	–	–
	12	–	–	–	–	–	–
	16	–	–	–	–	–	–
	20	–	–	–	–	–	–
	24	–	–	–	–	–	–
HvCO1	0	–	–	–	–	–	–
	4	a	a	b	a	a	b
	8	a	b	b	a	b	b
	12	a	b	ab	–	–	–
	16	–	–	–	–	–	–
	20	–	–	–	–	–	–
	24	–	–	–	–	–	–
HvCO2	0	–	–	–	–	–	–
	4	–	–	–	–	–	–
	8	–	–	–	–	–	–
	12	–	–	–	–	–	–
	16	–	–	–	–	–	–
	20	–	–	–	–	–	–
	24	–	–	–	–	–	–
HvFT1	0	a	b	b	a	b	b
	4	a	b	b	a	b	b
	8	a	b	b	a	b	b
	12	a	b	b	a	b	b
	16	a	b	b	–	–	–
	20	a	b	b	–	–	–
	24	a	b	b	–	–	–
Ppd-H1	0	a	b	b	–	–	–
	4	–	–	–	–	–	–
	8	–	–	–	–	–	–
	12	–	–	–	–	–	–
	16	–	–	–	–	–	–
	20	a	b	b	a	b	ab
	24	a	b	ab	–	–	–
HvCCA1	0	–	–	–	–	–	–
	4	a	b	ab	–	–	–
	8	a	b	b	a	b	b
	12	a	ab	b	–	–	–
	16	a	a	b	a	ac	bc
	20	–	–	–	–	–	–
	24	a	ac	bc	a	ac	bc
HvTOC1	0	–	–	–	–	–	–
	4	–	–	–	–	–	–
	8	–	–	–	–	–	–
	12	–	–	–	–	–	–
	16	–	–	–	–	–	–
	20	–	–	–	–	–	–
	24	–	–	–	–	–	–

a-c: Distinct letters indicate significant differences, –: No significant difference

Supplemental Materials and Methods

Preparation of shoot apical meristems (SAMs)

The two-row spring barley (*Hordeum vulgare* L.), cv. Nishinohoshi, served as the plant material. Seeds underwent sterilization in 2% (w/v) sodium hypochlorite for 30 minutes and incubation overnight at 4˚C in darkness. SAMs were obtained by eliminating the coleoptile and the first three leaves from the hydrated seeds utilizing a NanoPass 34-gauge needle (ϕ 0.2 mm; TERUMO, Japan) as outlined by Imai et al. (2020). The exposed SAM embryos were subsequently dissected from the seeds and positioned in the center of a Murashige and Skoog (MS) medium enriched with maltose (30 g/L), 2-morpholinoethanesulfonic acid (MES) monohydrate (0.98 g/L, pH 5.8), a plant preservative mixture (3%, v/v; Nacalai Tesque, Japan), and phytagel (7.0 g/L; Sigma Aldrich, USA). Approximately 30 to 40 embryos were organized in circles at the center of the MS medium for particle bombardment.

Introduction of CRISPR/Cas9 ribonucleoproteins to SAMs

Gold particles were coated with CRISPR/Cas9 ribonucleoproteins essentially as previously described (Kumagai et al., 2022). Two gRNAs targeting *Ppd-H1* (HORVU.MOREX.r3.2HG0107710.1), gRNA1 and gRNA2, were synthesized via the Precision gRNA Synthesis Kit (Invitrogen, USA). Template oligomers for each gRNA can be found in Supplemental Table S1. CRISPR/Cas9 RNPs were assembled by incubating 10 µg of each gRNA and spCas9 in a 20 µL CutSmart buffer (New England Biolabs, USA) at ambient temperature for 10 minutes. Five microliters of TransIT-LT1 transfection reagent (Takara Bio, Japan) was added to the RNP solution and allowed to incubate at room temperature for an additional 5 minutes. The RNPs were combined with 2.7 mg of 0.6 µm gold particles (Bio-Rad, USA) and incubated at room temperature for 10 minutes. The resultant gold particle suspension, coated with RNPs, was

spread on a hydrophilic film (3M, USA) to dry. These gold particles were introduced to SAMs at 1,350 psi using the PDS-1000/He system (Bio-Rad).

Screening for genome edited plants

Post RNP introduction, SAMs were cultivated on the aforementioned MS medium for two weeks, then transitioned to soil. Fifth leaves from E_0 plants were isolated for genotyping through cleaved amplified polymorphic sequences (CAPS). *Ppd-H1* partial fragments encompassing gRNA1 or gRNA2 target regions were amplified with gene-specific primers, as noted in Supplemental Table S1. The polymerase chain reaction utilized KOD One (Toyobo, Japan). The following conditions were used for amplification of partial *Ppd-H1* fragments: 98 ◦C for 30 s, followed by 32 cycles of 98 ◦C for 10 s, 60 ◦C for 5 s, and 68 ◦C for 5s. Resultant fragments were subjected to digestion by *Pvu* II or *Nla* III enzymes to identify mutations at the gRNA1 or gRNA2 target region, respectively. Undigested fragments observed via agarose gel electrophoresis were cloned into pCR-Blunt II-TOPO vector included in the Zero Blunt TOPO PCR cloning kit (Invitrogen) for sequencing. The genotype of the E_1 generation was discerned in a similar manner, using E_1 seedlings derived from individual E_0 plants.

Expression analysis

For circadian expression analysis, total RNA was extracted from mature 14-day-old leaves of plants cultivated under long-day (LD) conditions (16h light at 22˚C/8h dark at 15˚C). First-strand cDNA synthesis was achieved from 500 ng total RNA using the PrimeScript RT reagent kit (Takara Bio). Reverse transcription quantitative PCR (RT-qPCR) procedures employed the AriaMx Real-Time PCR System (Agilent, USA) with TB Green Ex TaqII (Takara Bio), normalizing gene expression relative to HvActin levels. Data delineates the mean ± SE with four biological replicates (n = 4). The primers for RT-qPCR are detailed in Supplemental Table S1.

For day growth expression analysis, RNA was extracted from mature leaves of plants at 7, 14, and 21 days after-germination (DAG), grown under the same LD conditions. Sampling took place 12 hours post-light activation. cDNA synthesis and RT-qPCR followed the protocols mentioned earlier. Data delineates the mean ± SE with four biological replicates (n = 4).

The phenotypic analysis of the *ppd-H1* mutants

Both *ppd-H1* mutants and wild-type seeds underwent sterilization in 2% (w/v) sodium hypochlorite for 30 minutes, followed by dark incubation at 4˚C. Germinated seeds were individually planted in 2-liter soil containers and cultivated in controlled growth rooms with LD conditions (16h light at 22˚C/8h dark at 15˚C). All plants received a 2000-fold diluted HYPONeX (HYPONeX JAPAN, Japan) solution weekly. The heading date was noted upon the appearance of the spike tip from the leaf sheath. Grass yield assessment involved harvesting the above-ground portion immediately prior to inflorescence development and drying at 85˚C for 7 days; the wild type and mutants were harvested at either 31 or 71 DAG. Grain yield measurements encompassed spike and seed counts, along with seed dry weight per plant. All of phenotypic analyses were performed with six biological replicates (n = 6).

Supplemental References

Imai, R., Hamada, H., Liu, Y., Linghu, Q., Kumagai, Y., Nagira, Y., Miki, R., Taoka, N. (2020). *In planta* particle bombardment (iPB): A new method for plant transformation and genome editing. Plant Biotechnology, 37(2), 171–176. doi.org/10.5511/plantbiotechnology.20.0206a.

Kumagai, Y., Liu, Y., Hamada, H., Luo, W., Zhu, J., Kuroki, M., Nagira, Y., Taoka, N., Katoh, E., Imai, R., 2022. Introduction of a second "Green Revolution" mutation into wheat via in planta CRISPR/Cas9 delivery. Plant Physiol. 188, 1838–1842. doi:10.1093/plphys/kiab570
